# High Plasma Cystine Levels Are Associated with Blood Pressure and Reversed by CPAP in Patients with Obstructive Sleep Apnea

**DOI:** 10.3390/jcm10071387

**Published:** 2021-03-30

**Authors:** Raphael Boneberg, Anita Pardun, Lena Hannemann, Olaf Hildebrandt, Ulrich Koehler, Ralf Kinscherf, Wulf Hildebrandt

**Affiliations:** 1 Department of Medical Cell Biology, Institute for Anatomy and Cell Biology, University of Marburg, Robert-Koch-Str. 8, 35032 Marburg, Germany; r.boneberg@googlemail.com (R.B.); norman-pardun@hotmail.de (A.P.); lena0712@web.de (L.H.); ralf.kinscherf@staff.uni-marburg.de (R.K.); 2 Department of Sleep Medicine, Division of Pneumology, Internal Medicine, University Clinics of Marburg, UKGM, Baldingerstr. 43, 35043 Marburg, Germany; olaf.hildebrandt@med.uni-marburg.de (O.H.); koehleru@med.uni-marburg.de (U.K.)

**Keywords:** sleep, breathing-disorder, intermittent hypoxia, oxidative stress, glutathione, cardiovascular risk

## Abstract

Obstructive sleep apnea (OSA) independent of obesity (OBS) imposes severe cardiovascular risk. To what extent plasma cystine concentration (CySS), a novel pro-oxidative vascular risk factor, is increased in OSA with or without OBS is presently unknown. We therefore studied CySS together with the redox state and precursor amino acids of glutathione (GSH) in peripheral blood mononuclear cells (PBMC) in untreated male patients with OSA (apnea-hypopnea-index (AHI) > 15 h^−1^, *n* = 28) compared to healthy male controls (*n* = 25) stratifying for BMI ≥ or < 30 kg m^−2^. Fifteen OSA patients were reassessed after 3–5-months CPAP. CySS correlated with cumulative time at an O_2_-saturation <90% (Tu90%) (r = 0.34, *p* < 0.05) beside BMI (r = 0.58, *p* < 0.001) and was higher in subjects with “hypoxic stress” (59.4 ± 2.0 vs. 50.1 ± 2.7 µM, *p* < 0.01) defined as Tu90% ≥ 15.2 min (corresponding to AHI ≥ 15 h^−1^). Moreover, CySS significantly correlated with systolic (r = 0.32, *p* < 0.05) and diastolic (r = 0.31, *p* < 0.05) blood pressure. CPAP significantly lowered CySS along with blood pressure at unchanged BMI. Unexpectedly, GSH antioxidant capacity in PBMC was increased with OSA and reversed with CPAP. Plasma CySS levels are increased with OSA-related hypoxic stress and associated with higher blood pressure. CPAP decreases both CySS and blood pressure. The role of CySS in OSA-related vascular endpoints and their prevention by CPAP warrants further studies.

## 1. Introduction

Obstructive sleep apnea syndrome (OSA) denotes recurrent episodes of apnea or hypopnea through complete or partial pharyngeal collapse leading to hypoxemia- reoxygenation cycles, i.e., chronic intermittent hypoxia (CIH). Together with concomitant pCO_2_ and intrathoracic pressure sways, CIH forms a unique pathophysiologic pattern involving chemoreceptor driven sympathetic arousals with sleep fragmentation, neurohumoral changes, and inflammatory/oxidative stress, all of which may convey cardiovascular and metabolic risks not attributable to prolonged nocturnal hypoxia alone [1,2,3,4,5,6,7]. Numerous observational studies and several interventional trials evaluating continuous positive airway pressure (CPAP) therapy have identified OSA as an independent cardiovascular factor of mortality and cardiovascular endpoints [8,9,10,11], including endothelial dysfunction [6], hypertension [12,13,14], stroke [11,15], coronary heart disease [16,17], and carotid plaque development [18]. However, prevention through CPAP is often incomplete, partly due to limited adherence/tolerance under “real-world” conditions [19,20]. This is also true for the more recently established OSA-associated risk of insulin resistance (IR), which is only reversed with supervised 8 h, but not with 4 h of CPAP per night [4,12].

There is a well-established close interrelation between OSA and obesity (OBS): According to community-based studies, the prevalence of moderate–severe OSA (AHI > 15/h) is 17.4% in males (9.1% in females) aged 50–70 years (data between 2008–2010), however, it reaches a dramatic 29.9% (13.9%) or even 56% (33.4%) in strata with BMI of 30–40 or > 40 kg m^−2^, respectively [21,22,23]. Thereby, OSA and OBS share common vascular risk profiles [5,6,12,24], i.e., endothelial dysfunction, hypertension, inflammation (CRP), IR, and dyslipidemia involving common inflammatory pathways such as NFκB activation with expression of TNF, IL6, IL1β, or adhesion molecules in endothelial or peripheral mononuclear cells (PBMC), as well as oxidative stress markers like decreased endothelial eNOS expression and phosphorylation or increased nitrotyrosines [25,26,27,28,29,30,31,32,33,34,35,36]. Selected OSA-related triggers related to CIH are unique including, e.g., VEGF expression [37,38], and, accordingly, some proatherogenic factors may respond differentially to CPAP and weight loss or their combination [12], such as CRP. Yet, the identification of the separate vascular risk mediators with OSA or with OBS has remained a challenge to clinical studies [5,39,40].

The plasma cystine level (CySS) is a relatively novel independent vascular risk factor of endothelial dysfunction and arterial stiffness in healthy subjects [41,42] that has recently been shown to predict mortality in patients with coronary artery disease [43]. In fact, a CySS increase of one standard deviation is associated with a 26% increase in mortality after adjustment for multiple traditional risk factors. CySS is the disulfide of the thiol compound cysteine (Cys), which is 8–10 times less concentrated in the plasma. Together, Cys and CySS constitute the most abundant extracellular thiol redox couple, which may convey non-radical-based pro-oxidative signals to endothelial cells, PBMC, or other cells leading to expression of the above-mentioned inflammatory cytokines and adhesion molecules [44,45,46].

Whether OSA is independently associated with increases in CySS and related risks is presently unknown. A previous study had reported significant increases of the total plasma ‘Cys’ in patients with mild to severe OSA patients [47]. These measurements, however, represented the redox couple Cys and CySS in total, including the albumin-bound CySS-fraction (i.e., the ‘Cys’ concentration ranged between ca 400 and 500 µM) and, therefore, provided no information on the acid-soluble plasmatic fraction of the disulfide CySS, as the relevant vascular risk factor (ranging between 40–110 µM) [43,48,49,50]. Additionally, the fraction of the putatively counteracting, i.e., protective effect of the thiol-containing Cys remained unclear [46,48,49,50]. Moreover, the group of OSA patients were on medication and had a significantly, by 4 kg m^−2^, higher BMI, which could have contributed to higher CySS [44,46].

We presently hypothesized that increases in acid-soluble plasma CySS with OSA could be detected when comparing untreated OSA patients (AHI > 15 h^−1^) to healthy controls with control for BMI and, furthermore, that high CySS might represent a novel link between OSA and related vascular risks like blood pressure (BP) increases. This hypothesis was challenged by studying the intraindividual effect of home-based CPAP therapy on CySS and BP in the absence of weight changes. In addition, we explored differences between groups with and without cumulative hypoxic stress (cHPX), to evaluate the impact of “intensity” beside that of “frequency” of OSA-/CIH-related episodes on CySS and BP.

## 2. Materials and Methods

### 2.1. Subjects and Study Design

**Figure 1:**
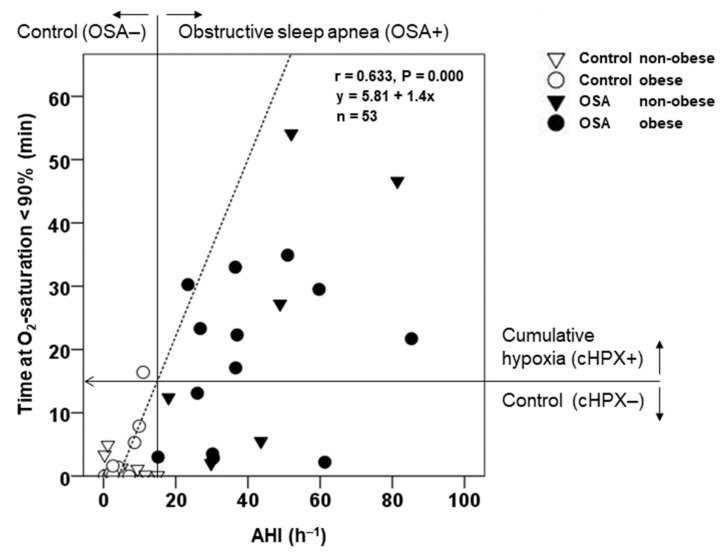
Correlation between individual cumulative time at O_2_-saturation < 90% (Tu90%) and the individual apnea-hypopnea-index (AHI). The graph presents the regression line and indicates the cut-off values of AHI > 15 h^−1^ (vertical line) for diagnosis of moderate to severe obstructive sleep apnea syndrome (OSA) and the corresponding cut-off of Tu90% > 15.2 min (horizontal line) defining cumulative hypoxic stress (cHPX) as an explorative group assignment (see also ‘Method’ statistics section). Using this cHPX cut-off, 7 OSA patients are newly assigned to controls (without cHPX), while one OSA control subject with AHI < 15 h^−1^ is newly assigned to the cHPX group. Note that for better differentiating of values around the two cut-offs, only values of Tu90% below 60 min are shown, while r, *p*, and the regression line within the graph represent the total study population (*n* = 53).

We presently compared CySS and BP (primary outcome) as well as intracellular (PBMC) glutathione and its other intra- and extracellular precursor amino acids (secondary outcome) between a group of 28 non-smoking, untreated, male patients with untreated moderate to severe OSA (AHI > 15 h^−1^) to a group of 25 non-smoking similarly aged healthy control subjects (with exclusion of OSA) with stratification for BMI ≥30 (obese) or < 30 (non-obese) kg m^−2^ (cross-sectional design, see Table 1; for explorative assignment to groups with and without cHPX, see Figure 1 and Table 2). Furthermore, we evaluated the effect of 3–5 months of home-based CPAP in 15 OSA patients (longitudinal design). All subjects gave their informed oral and written consent to participate in the study, which was approved by the local Ethical Committee of the Medical Faculty (FB20) of the University of Marburg (63/11, 12 September 2011) and was performed according to good laboratory and medical practice (GLP, GMP) and to the amended declaration of Helsinki (1996). Any study-related delay in CPAP-therapy initiation was excluded by individual adjustments of time schedules for eligible outpatients of the Department of Sleep Medicine of the University Clinics of Marburg. All subjects underwent an initial stationary two-nights-polysomnography for exclusion or diagnosis of OSA. The subjects’ initial medical assessment furthermore included their medical history, physical examination, routine laboratory venous blood parameters, bilateral brachial BP measurement in duplicate using the validated automated visomat comfort device (Uebe Medical GmbH, Külsheim, Germany) after 20 min of semireclined rest [51], as well as ultrasonic measurement of carotid intima-media-thickness (cIMT) by the Logiq E9 system and 9D-L-scanner (GE, Connecticut, USA), pulmonary function, and a 12-lead electrocardiogram (ECG) at rest. Main exclusion criteria were: Age < 35 or > 65 years, smoking or history of smoking, any medication, any vitamin, Cys or n-acetylcysteine supplementation, central or mixed sleep apnea, prior CPAP treatment, type 2 diabetes mellitus or HbA1c > 7.0, known arterial hypertension or measured systolic or diastolic brachial arterial BP > 180 and > 115 mmHg after 20 min rest, any known cardial, pulmonary, or other internal, neurological, or psychiatric, immunological or inflammatory disease, alcohol or drug abuse; missing oral or written consent.

### 2.2. Inpatient Polysomnography

All participants underwent a polysomnography on two consecutive nights at the nationally licensed Department of Sleep Medicine, University Clinics of Marburg using the SIDAS-GS-System (Stimotron Medizinische Geräte GmbH, Hamburg, Germany). The study data were obtained during the second night, while the first night allowed for the subjects’ familiarization with the equipment and the environment. The measurements comprised: AHI (during total sleep time (TST)) of obstructive, central nervous or mixed origin, arousal index (n/h TST) mean and minimal SaO_2_ in REM or non-REM sleep phases, time (min) spent at SaO_2_ < 90%, < 80%, or < 70%, and time (min) fraction (%) sleep stages REM and non-REM (1, 2 or 3, 4). Initiation and individual adjustment of CPAP therapy for home-based treatment in OSA patients were completed during night 3 or 4 at the sleep center. After 3–5 months of home-based CPAP, OSA patients were re-hospitalized for evaluation and individual readjustment of CPAP levels and assessment of compliance to CPAP (data stored within the device).

### 2.3. Blood Parameters

Postabsorptive blood samples were drawn from an antecubital vein in overnight-fasted subjects after 15 min rest between 7:00 and 9:00. Routine laboratory parameters included plasma levels of triglycerides, total cholesterol, low-density-lipoprotein (LDL), high-density lipoprotein (HDL), HbA1c, glucose, insulin, and the Homeostasis Model Assessment index for IR (HOMA-IR, i.e., “insulin (µU/mL) × glucose (mg/100 mL)/405”). The total plasma homocysteine was measured via enzymatical formation and metabolization of cystathionine with NADH-based detection of pyruvate formation (Beckmann Coulter Inc., Brea, CA, USA). Plasma levels of acid-soluble of CySS, glutamate, and glycine were determined in plasma samples from heparinized blood after deproteinization with sulfosalicylic acid (SSA, 50%) using high-performance liquid chromatography (HPLC) technique (Biochrom 30plus, Onken, Gründau, Germany). Moreover, PBMCs were isolated from phosphate-buffered EDTA-blood by means of Histopaque density gradient ultracentrifugation (Sigma, Munich, Germany) according to the manufacturer’s instruction. After precipitation with SSA, sonification, and centrifugation, the supernatant was used for determination of total and reduced GSH as well as GSSG content by Tietze’s assay [52]. The intracellular protein concentration of PBMC as assessed by the colorimetric Biorad protein assay (Biorad Labortories, Munich, Germany) was used for normalization of intracellular content of amino acids, GSH, or GSSG.

### 2.4. Statistics

Continuous variables were described as mean ± standard error of the mean (SEM) for the total group of controls and OSA patients (before and after CPAP) as well as their non-obese and obese strata. Two-factorial ANOVA was applied to the total group under test for cross-sectional analyses of the separate impact of OSA, OBS, or their interaction. Significant differences between groups or strata with and without OSA (indicated by *) or those with and without OBS (indicated by #) were (post hoc) detected by the unpaired Student’s t-test. Intraindividual (longitudinal) effects of CPAP were analyzed by the Student’s t-test for paired observation. Bivariate correlations between individual measures (e.g., CySS and systolic or diastolic BP) were presented as scatter plots with indications of group and strata, regression line, Pearson’s correlation coefficient r, and the *p*-values. Multivariate regression with and without adjustment for the factor age (independent variable) was used to analyze the impact of Tu90% or BMI (independent variables) on the primary outcome CySS (dependent variable) or the impact of CySS (independent variable) on diastolic or systolic BP (dependent variable).

Since the diagnose of moderate-severe OSA based on AHI > 15 h^−1^ refers to the “frequency” of apnea-hypopnea episodes, we additionally aimed to explore the impact of “hypoxic stress” on the main outcome variables. Therefore, a group exposed to cumulative hypoxic stress (cHPX) was defined by the cumulative time spent at SaO_2_ < 90% (Tu90%) and compared to its control group. The rationale for the explorative cut-off of Tu90% for the cHPX group is illustrated in Figure 1: Within an expected correlation between Tu90% and AHI, an explorative Tu90% cut-off (> 15.2 min within the present study population, see Results and horizontal line in Figure 1) corresponds to a common AHI cut-off of > 15 h^−1^ (vertical line: OSA group on the right, control group on the left). The application of the Tu90% cut-off for a cHPX group led to assignment of 8 OSA patients to the control (Tu90% < 15.2 min), and of 1 control subject without OSA to the cHPX group. Thus, the present comparison between OSA group and its control group included 28 and 25 subjects, respectively (Table 1), while that between cHPX group and its respective control group was involved 21 and 32 subjects, respectively (Table 2).

A *p*-level < 0.05 was considered as statistically significant. No Bonferroni-inspired correction was intended given the explorative approach within this hypothesis-generating study. The SPSS-software version 22.0 (IBM, Munich, IBM, Munich, Germany) was used for all statistical procedures.

## 3. Results

### 3.1. Epidemiology, Polysomnography, and Cardiovascular Risk Profile at Baseline

The mean ± SEM values for the common OSA diagnosis by AHI > 15 h^−1^ are presented in Table 1, while those for the explorative cHPX assignment by Tu90% > 15.2 min are given in Table 2 (for details see the Method statistics section). The subjects’ age was comparable between obese OSA patients (Table 1) and obese or non-obese controls, however, as a study limitation, > 4 years of recruitment of eligible non-smoking, unmedicated, non-obese OSA patients in this mono-centered study resulted in a small stratum with significantly higher age of OSA patients compared to their controls (*p* = 0.004, Table 1) or of the cHPX group compared to its control (*p* = 0.038, Table 2). BMI was comparable between the OSA patients (as recruited) and their controls or their non-obese strata, and differed only marginally (by 1.2 kg m^−2^) between the obese strata of OSA and controls (*p* < 0.05, Table 1). However, explorative assignment to cHPX (Table 2) yielded a significantly by 3.5 kg m^−2^ higher BMI of the cHPX group compared to the relevant controls.

In line with the exclusion criteria, the non-smoking study participants had normal pulmonary function parameters, and there was now significant difference between the OSA group and the control group with regard to vital capacity (VC) (5.12 ± 0.21 vs. 5.49 ± 2.47, respectively), relative VC (108.8 ± 4.5% vs. 112.3 ± 4.0%), forced expiratory volume within 1st s (FEV1) (4.04 ± 0.16% vs. 4.49 ± 0.21%), relative FEV1 (106.5 ± 4.1% vs. 112.2 ± 4.7%), FEV1%VC (79.4 ± 1.3% vs. 81.8 ± 1.2%), relative FEV1%VC (101.3 ± 1.5% vs. 102.1 ± 2.2%), or maximal expiratory flow at 50% expiration (MEF50%) (4.76 ± 0.31 L/s vs. 5.60 ± 0.36 L/s).

Patients diagnosed with moderate to severe OSA (AHI > 15 h^−1^) had a significantly higher AHI (TST) (48.8 h^−1^, range 15.1–94.2 h^−1^) than controls (5.0 h^−1^, range 0.3–15.0 h^−1^) with comparable values between their obese or non-obese strata (Table 1). Likewise, the explorative cHPX group showed significantly higher AHI compared to controls with no significant difference between obese and non-obese strata (Table 2). Tu90% amounted to 61.8 min in OSA patients and to 81.0 min in the cHPX group as opposed to 1.9 min and 2.4 min in their respective control group (*p* < 0.001) (Table 1 and Table 2). As shown in Figure 1, there was a significant close correlation between Tu90% and AHI (r = 0.63, *p* < 0.001), which was used to define the explorative assignment of subjects to a cHPX group (see the Methods statistic section). Mean peripheral O_2_-saturation (averaged over TST) was moderately but significantly lower in OSA patients (91.5%) or in the cHPX group (91.8%) compared to the respective controls (94.3% or 94.4%), and similar between obese and non-obese strata (Table 1 and Table 2). Heart rate tended to be higher in the total group of OSA patients or the cHPX group compared to controls, a significant difference was, however, only detectable between the obese strata. Systolic and diastolic BP were found to be significantly higher with OSA or cHPX compared to controls, whereby ANOVA detected a (borderline-)significant impact of both OSA or cHPX and OBS, but no significant interaction (Table 1 and Table 2). Notably, in contrast, a significantly higher cIMT was associated with OSA or cHPX, but not with OBS. This was also true for HbA1c, fasted plasma glucose levels, and for HOMA-IR (with OSA only) within the non-diabetic range (HbA1c ≤ 7%). While HDL, LDL, and total cholesterol were unaffected by OSA or OBS, triglycerides showed a significant impact of OSA (Table 1), which was mainly due to significantly higher values in the non-obese OSA patients compared to controls, not found within the cHPX group (Table 2). Among non-traditional vascular risk factors, homocysteine was found to be within the normal to moderately elevated range, with slightly but significantly lower values observed with OSA, but not with cHPX (Table 1 and Table 2). Circulating levels of TNF were found to be comparable between groups and strata with OSA and cHPX (Table 1 and Table 2).

### 3.2. Amino Acids and Glutathione Levels at Baseline

**Figure 2:**
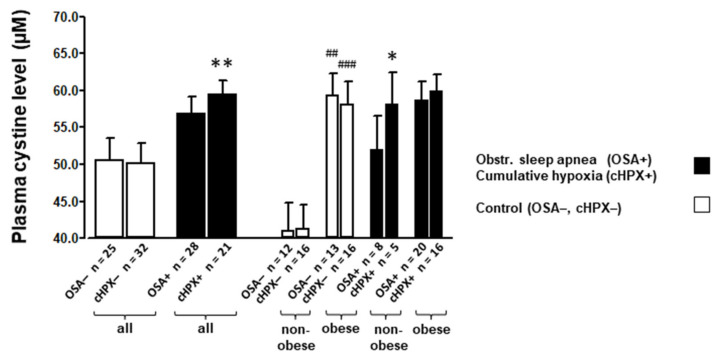
Mean (±SEM) plasma cystine (CySS) levels of OSA patients (AHI > 15 h^−1^) or the explorative group with cumulative hypoxic stress (cHPX, defined as time at O_2_-saturation < 90% > 15.2 min) compared to their respective controls group with stratification for non-obese and obese BMI. According to a two-factorial ANOVA, both cHPX and OBS significantly and independently impacted plasma CySS levels (see also Table 1 and Table 2). * for *p* < 0.05, ** *p* < 0.01 for significant differences between OSA patients or the cHPX group (total group or strata) and their respective control (strata); ## for *p* < 0.01, ### for *p* < 0.001 for significant differences between obese and non-obese controls as detected by unpaired *t*-test.

Expectedly, within the total study population, the plasma CySS levels were found to be significantly impacted by OBS (*p* < 0.001 by ANOVA) with significantly by 44.6% higher concentrations in the obese vs. non-obese controls (59.3 ± 3.0 vs. 41.0 ± 3.8 µM), whereas OSA (*p* = 0.164 by ANOVA) defined as AHI > 15 h^−1^ had no significant impact (Table 1, Figure 2). However, with explorative assignment of subjects to the cHPX group defined as Tu90% > 15.2 min (Table 2, Figure 2), a significant and independent effect of cHPX (*p* = 0.016 by ANOVA) on CySS besides that of OBS (*p* < 0.006, by ANOVA) was detected (together with a significant interaction of both). Plasma CySS plasma levels were significantly higher within the total cHPX group compared to controls (59.4 ± 2.0 vs. 50.1 ± 2.7 µM, *p* < 0.01) (Table 2, Figure 2). Moreover, a significant increase in plasma glutamate levels was found with both, the OSA (Table 1) and cHPX (Table 2) with indication of a significant interaction of OBS, which independently of OSA impacted plasma glutamate levels. Plasma glutamate and CySS levels were highly significantly correlated (r = 0.636, *p* < 0.001). In terms of percentage, the increases in plasma CySS and glutamate levels were 12.1% and 30.0% in OSA patients (Table 1), respectively, and 18.6% and 36.1% (Table 2) in the cHPX group compared to their respective controls. In contrast, no such effects were found with OSA or cHPX regarding the plasma level of glycine, i.e., another GSH precursor (Table 1 and Table 2). Regarding intracellular (PBMC) amino acid levels, notably, CySS was not detectable, and, furthermore, levels of glutamate and glycine within this compartment were not significantly affected by OSA, cHPX, or OBS (Table 1 and Table 2).

Total GSH and reduced GSH, as well as GSSG within PBMC, were determined within sample pools per stratum of OSA patients and their control excluding statistical analyses (Table 1). Contrary to expectation, no limitation or pro-oxidative shift within this predominant intracellular thiol redox couple could be detected; rather, total and reduced GSH tended to be higher with OSA (total group or non-obese stratum) as well as with OBS (obese vs. non-obese stratum in the control group).

### 3.3. Correlation of CySS and Glutamate to BMI, AHI, and BP

**Figure 3:**
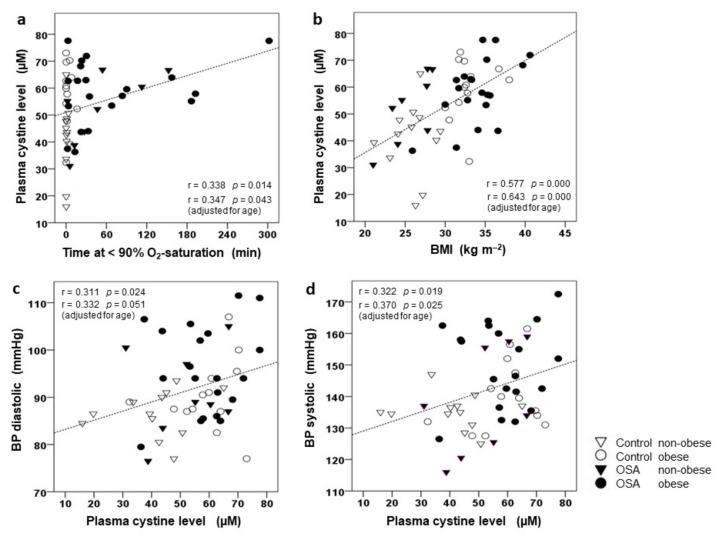
Correlation of plasma cystine (CySS) concentration to cumulative time spent at O_2_-saturation < 90% (Tu90%) (**a**) and to BMI (**b**) as well as correlation between diastolic (**c**) and systolic (**d**) brachial blood pressure (BP) and plasma CySS concentration. Graphs present scatterplots of individual values with indications of groups (OSA, control) and their strata (non-obese, obese), the regression lines as well as regression coefficients r and *p*-values. In addition, r and *p*-values are given for (multivariate) correlation adjusting for age.

Plasma CySS levels showed a significant positive correlation with both, Tu90% (but not AHI) and BMI (Figure 3a,b, respectively) within the total study population. This correlation was only marginally strengthened when adjusting for age, i.e., when controlling for the small but significantly higher age of the (non-obese) OSA patients compared to controls. Notably, there was no significant (bivariate or multivariate) correlation between age and CySS. Importantly, we found a significant and positive correlation of diastolic and of systolic BP to CySS (Figure 3c,d, respectively) with or without adjustment for age. No such relation existed between CySS and cIMT, parameters of glycemic control (HbA1c, fasting plasma levels of glucose and insulin, or HOMA-IR), plasma lipids, or homocysteine. Moreover, plasma levels of glutamate were found to be significantly and positively related to Tu90% (r = 0.467, *p* < 0.01) or AHI (r = 0.380, *p* < 0.011) and BMI (r = 0.383, *p* < 0.01). In addition, there was a significant relation of diastolic (r = 0.298, *p* < 0.05) and systolic (r = 0.332, *p* < 0.05) BP to glutamate, while cIMT, parameters of glycemic control, plasma lipids, or homocysteine were unrelated to this amino acid. In contrast, plasma glycine levels were found to be unrelated to any of these parameters.

### 3.4. Effect of CPAP on CySS and Cardiovascular Risk Factors

**Figure 4:**
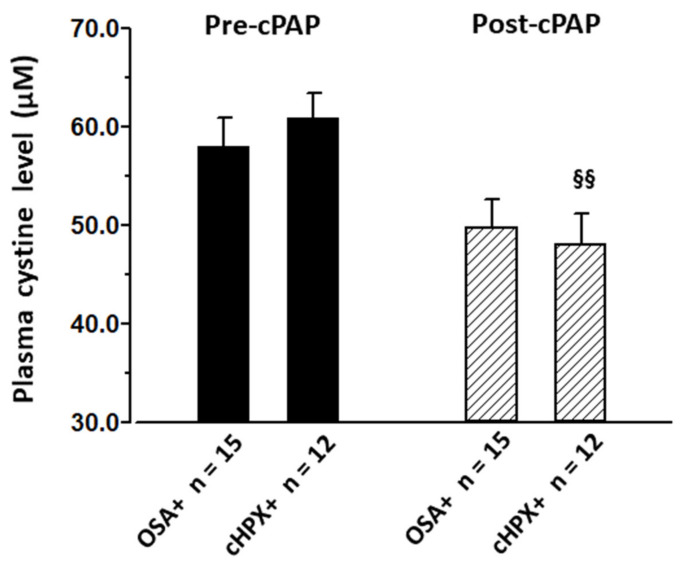
Plasma cystine (CySS) plasma levels before and after 3–5 months CPAP therapy in OSA patients (AHI > 15 h^−1^) as well as in the explorative cHPX group (Tu90% > 15.2 min) (see “Methods” section “statistics”). Data are presented as mean (± SEM); §§ for *p* < 0.01 by *t*-test for paired observations (post- vs. pre-CPAP values).

3–5 months of home-based CPAP in OSA patients (*n* = 15, AHI 47.9 h^−1^, Tu90% 73.0 min) and in the explorative cHPX group (*n* = 12, AHI 52.1, Tu90% 89.8 min) with mean CPAP adherence (compliance) of 5.1 ± 0.7 h and 5.2 ± 0.7 per night, respectively, effectively reduced AHI or Tu90% (Table 3), interestingly to below control levels (Table 1 and Table 2). Thereby, CPAP yielded virtually no changes (improvements) in BMI, lipid profiles, glycemic control, or homocysteine, irrespective of subjects’ assignment to OSA or cHPX. In contrast, CySS, as well as glutamate and glycine, were reduced by CPAP, notably, to below control levels (Table 3, Figure 4). In the case of CySS, this decrease with CPAP reached significance only within the cHPX group with pre-treatment Tu90% > 15.2 min, whereas glutamate and glycine were decreased irrespective of the subjects’ assignment to OSA or cHPX. Importantly, there were concomitant decreases in diastolic and systolic BP, which—like that of CySS—only reached significance within the cHPX group. Thereby, pre-to-post-CPAP changes in systolic or diastolic BP were not significantly related to those in CySS, glutamate, or glycine. Moreover, mean values (pooled samples) of total and reduced GSH and those of GSSG were found to be decreased with CPAP, i.e., CPAP partly reversed the OSA- and OBS-related trends towards elevation in GSH, but not in GSSG.

## 4. Discussion

The present study shows for the first time that the plasma CySS level, a novel important vascular risk factor [41,42,43], is significantly and independently increased not only with OBS, but also with a certain degree of OSA-related cumulative hypoxic stress (cHPX, presently defined as Tu90% < 15.2 min) rather than a certain frequency of episodes (AHI > 15 h^−1^, used for OSA diagnosis). The difference in mean CySS of 9.2 µM (see Table 2) between the cHPX group (*n* = 21) and its control group (*n* = 32) was detected with power of ≥ 80% (*p* < 0.05) at normal CySS distribution (minimal group size of *n* = 21 required). In line with this cross-sectional finding by two-factorial ANOVA, plasma CySS was significantly related to both BMI and Tu90%, but not to AHI. As evidence for a causal role of OSA-related cHPX in such plasma CySS increases, we found significant decreases in CySS with CPAP treatment in the cHPX group, notably, at unchanged BMI and other vascular risk factors including glycemic marker, plasma lipids, homocysteine, and circulating TNF. This interventional finding, though not controlled by a sham-treatment, may somewhat argue against a bias possibly arising from a slightly but significantly higher BMI in the cHPX group (Table 2) compared to the control group. As besides CIH-related (cumulative) hypoxic stress, CPAP prevents several other aspects within the unique pathophysiological pattern with OSA, like intrathoracic pressure and blood pCO_2_ sways, or sympathetic and neurohumoral activation, the exact trigger for CySS increases and its therapeutic reduction has to remain open.

The present study, in addition, confirms a close independent association of high CySS with OBS [44,46], notably within a range of non-morbid obesity and with indication for an interaction with the effect of cHPX. In view of these results, undiagnosed OSA or cHPX may have been a likely confounder in previous studies on OBS and CySS without polysomnography. Though the subjects’ age, a likely confounder of CySS [49], was by 4.3 years higher in the non-obese OSA patients than in their control stratum, we presently detected no significant correlation between CySS and subjects’ age (r = 0.143, *p* = 0.31). Furthermore, adjustments for age had little effect on the correlations of CySS to Tu90% and BMI (Figure 3a,b) or to diastolic and systolic BP (Figure 3c,d).

As another important novel finding, diastolic as well as systolic BP, known and presently confirmed to be increased with both OSA and OBS [5], were significantly related to plasma CySS (with or without age-adjustment), and like CySS were significantly decreased with CPAP in the cHPX group, i.e., subjects experiencing considerable cumulative hypoxia (Tu90% > 15.2 min), unlike those with an OSA diagnosis (AHI > 15 h^−1^). The observed cross-sectional correlation between CySS (or glutamate) and blood pressure before CPAP treatment and their selective decrease through CPAP (at unchanged traditional vascular risk factors) may point at a possible role of CySS (or glutamate) in OSA-related hypertension warranting further dedicated larger studies.

Indeed, an increased plasma level of CySS has been identified (together with low plasma glutathione levels) as a novel, independent predictor of (cardiovascular) mortality and myocardial infarction in patients with coronary heart disease [43]. More relevant to the present study, it was shown in healthy non-smokers aged 20–70 years that CySS was significantly negatively correlated to endothelial function (i.e., endothelial-dependent, but not endothelium-independent, vasodilation) as an early endpoint of developing hypertension yielding strong prediction even after adjustment for the Framingham risk score and C-reactive protein [41]. A similar correlation has been reported between CySS and arterial elasticity [42]. As the main component (90%) of the most abundant extracellular thiol/disulfide (Cys/CySS) redox couple, CySS may convey specific non-radical based oxidative (aminothiol) signals to Cys residues of membrane proteins of both endothelial cells and circulating PBMC, resulting in proatherogenic/inflammatory activation, NO-deficiency, and endothelial dysfunction contributing to hypertension [41,44]. Increased plasma CySS levels with or without decreases in Cys have also been reported for low-grade inflammatory vascular risks factors like aging, hyperlipidemia, obesity [44,48,49,50,53], i.e., confounders that were presently addressed. In vitro, different types of monocytes (U937, THP1) show increased cell adhesion, NFκB activation, and upregulation e.g., of IL1β and P-selectine upon acute exposure to proxidative Cys to CySS ratios as extracellular stimuli [44]. As a non-radical oxidative stress biomarker, CySS may thus contribute to the inflammatory/oxidative responses of circulating PBMC as observed in patients with OSA, OBS, or other conditions [26,27,28,29,30,31,32,35,36,53]. Yet, CySS may potentially vary from other important indicators of radical-based oxidative damage, like oxLDL, in its response to CPAP with a compliance ranging above 4 h per night [54,55,56]. This point may require a sham-controlled interventional trial design regarding CPAP.

Presently, the cause of a significant, CPAP-reversible increase in extracellular/plasma CySS, glutamate, as well as of intracellular GSH with OSA remains unclear, but may involve a common adaptive mechanism, given the correlation between CySS and glutamate as GSH precursors: As a major regulator of antioxidative defense and survival pathways, the transcription factors nuclear factor erythroid 2-related factor 2 (NFE2L2, NRF2) is effectively activated (stabilized and translocated) through hypoxia and/or oxidative or nitrosative stress, even in large tissues like skeletal muscle [57,58,59]. Thereby, NRF2 not only upregulates the glutamate-cysteine-ligase catalytic and modifier subunit (GCLC, GCLM) as rate limiting for glutathione synthesis and GSH transporters [57,60], but also increases the expression of the light-chain subunit xCT (especially the 40 kDa isoform) of the ubiquitously expressed and sodium-independent cysteine-glutamate exchange system X_c_^−^ (SLC7A11), which critically regulates intracellular glutathione synthesis via CySS uptake in response to oxidative stress (but not hyperglycemia) [59,61]. NRF3 activation may thus potentially contribute to increases in GSH synthesis, which might still have remained limited by CySS ranging at very low, i.e., non-detectable intracellular levels. Such an adaptive antioxidative mechanism may help to maintain or increase GSH levels in several tissues in the presence of increased lipid oxidation, CRP, and NF-κB-activation with CIH (with clinically relevant mild O_2_-desaturation), at least in rats [28], and may be involved in the somewhat unexpectedly increased GSH-synthesis in PBMC of smokers or COPD patients [50,62,63].

The cause of increase in plasmatic CySS with cHPX and its reversal with CPAP remains speculative, as it may involve increased availability and oxidation of Cys via complex recycling pathways or delayed uptake (insulin–resistance). A simple pro-oxidative shift within the Cys/CySS redox couple may not explain a >15% increase in CySS (constituting ~90% of this redox couple as a disulfide of two Cys), however, an increased intracellular generation and cellular release of Cys has been suggested in the context of a CySS-Cys cycle, which is accelerated upon upregulation of the system X_c_^−^ (at little GSH changes) and considered to effectively contribute to extracellular NO availability by transfer, e.g., from S-nitrosothiols to smooth muscle [54,64,65]. In view of the significant correlation between plasma glutamate and CySS (r = 0.42, *p* = 0.001), it is also noteworthy that a high plasma glutamate level may be an effective inhibitor of CySS uptake via X_c_^−^ [64], contributing to relatively high extracellular/plasma CySS levels (putatively even at increased CySS uptake). Possible causes of increases in extracellular/plasma glutamate levels are also speculative at present, but may include increased release, e.g., via the above-mentioned upregulation of the X_c_^−^ system itself, or a downregulation of the glutamate importer GLAST (EAAT1), which can occur upon hyperglycemia as relevant to the present OSA group [61].

Due to its limited size, the present mono-centered study is considered hypothesis-generating, however, as a strength, it enrolled only unmedicated, non-smoking male subjects without common comorbidities except for blood pressure increases within a certain range (see exclusion criteria). The accordingly restricted eligibility of untreated non-obese OSA patients (stratum *n* = 8) led to moderate age-related bias, which we adjusted for by multiple regression. Our cross-sectional evaluation of the impact of cHPX on CySS was limited by a bias through a higher BMI in the cHPX group, arising from the explorative reassignment of OSA patients and controls. However, the CySS-lowering effect through CPAP intervention at unchanged BMI strengthened the present evidence for increased plasma CySS levels with OSA-related cHPX. Certainly, a conclusive study on the causal impact of cHPX on CySS and related vascular risks will require a controlled randomized CPAP intervention and should also address the oxygen desaturation index (ODI) to further characterize “hypoxic stress”. Regarding the also novel intracellular findings on GSH and GSSG, isolated PBMC comprises monocytes beside B- or T-cells, and may only partly represent cells involved in early atherogenesis. Moreover, it should be kept in mind that systemically circulating cells are physiologically exposed to alternating pO_2_, which is reminiscent of CIH and obviously are additionally challenged by the unique OSA-related stress.

## 5. Conclusions

In summary, our data suggest (generate the hypothesis) that OSA-related cHPX beside OBS is independently and significantly associated with increased plasma levels of CySS, which are reversed by CPAP and attributable in part to degree/duration rather than frequency of hypoxic episodes. As systolic and diastolic BP (in a normal to slightly elevated range) were significantly related to CySS and similarly responded to CPAP at unchanged BMI, glycemic marker, plasma lipids, homocysteine, or circulating TNF, our data may be indicative of an early non-radical based vascular risk through high plasma CySS with OSA-related cHPX in addition to that through OBS. As a strong predictor of endothelial dysfunction, CySS may contribute to OSA-related hypertension (beside other factors like peripheral chemoreceptors [5]), warranting evaluation of its role in the variable responsiveness of hypertension and other vascular risks to CPAP.

## Figures and Tables

**Table 1:** Cross-sectional comparison between OSA patients (AHI < 15 h^−1^) and controls with stratification for non-obese and obese BMI: Anthropometry, polysomnography, vascular risk factors, cystine, as well as glutamate, glycine, and glutathione.

		Control	OSA	Control		OSA		*p* (ANOVA)		
		Total	Total	Non-Obese	Obese	Non-Obese	Obese	OSA	OBS	Interact.
*n*		25	28	12	13	8	20	53	53	53
Age	(years)	44.5 ± 1.2	48.8 ± 1.3 *	43.5 ± 1.82	45.4 ± 1.6	52.6 ± 1.9 **	47.3 ± 1.5 ^#^	0.004	0.378	0.055
BMI	(kg m^−2^)	29.5 ± 1.2	31.6 ± 0.9	25.8 ± 0.7	32.8 ± 0.6 ^###^	25.6 ± 1.0	34.0 ± 0.7 ^###^*	0.333	<0.001	0.216
AHI	(h^−1^)	5.1 ± 0.8	48.8 ± 4.5 ***	5.3 ± 1.3	4.8 ± 1.0	43.8 ± 7.3 ***	50.8 ± 5.6 ***	<0.001	0.335	0.290
Tu90%	(min)	1.9 ± 0.86	61.8 ± 14.0 ***	1.02 ± 0.45	2.82 ± 1.43	51.6 ± 19.2 *	65.8 ± 18.3 **	0.001	0.470	0.542
Mean SaO_2_	(%)	94.3 ± 0.2	92.5 ± 0.36 ***	94.3 ± 0.2	94.3 ± 0.3	92.7 ± 0.5 *	92.3 ± 0.5 **	<0.001	0.465	0.443
Heart rate (at rest)	(min^−1^)	66.2 ± 2.1	70.9 ± 2.2	69.8 ± 3.3	63.0 ± 2.4	71.5 ± 5.2	70.7 ± 2.3 **	0.163	0.341	0.239
BP systolic	(mmHg)	137.9 ± 1.8	146.3 ± 2.8 *	135.1 ± 1.6	140.5 ± 3.1	138.1 ± 6.1	149.5 ± 2.9	0.081	0.006	0.210
BP diastolic	(mmHg)	88.6 ± 1.3	94.5 ± 1.8 *	86.5 ± 1.4	90.4 ± 2.1	91.0 ± 3.3	95.9 ± 2.1	0.041	0.018	0.470
carotid IMT	(mm)	0.62 ± 0.02	0.70 ± 0.03 *	0.60 ± 0.03	0.63 ± 0.03	0.72 ± 0.06	0.69 ± 0.03	0.024	0.719	0.559
HbA1c	(%)	5.51 ± 0.05	5.74 ± 0.08 *	5.49 ± 0.06	5.52 ± 0.09	5.73 ± 0.06 *	5.74 ± 0.11	0.037	0.720	0.951
Glucose (fasted)	(mg 100 mL^−1^)	92.6 ± 1.4	98.4 ± 2.2 *	93.3 ± 1.8	91.9 ± 2.2	98.3 ± 4.9 **	98.5 ± 2.5	0.049	0.891	0.718
Insulin (fasted)	(µU mL^−1^)	8.93 ± 1.91	14.16 ± 2.05	9.90 ± 4.10	9.24 ± 1.91	18.81 ± 7.09	13.98 ± 1.81	0.094	0.800	0.975
HOMA-IR	(---)	1.68 ± 0.28	3.57 ± 0.59 **	1.18 ± 0.21	2.15 ± 0.47	3.97 ± 1.79	3.42 ± 0.45	0.008	0.558	0.437
HDL	(mg 100 mL^−1^)	48.1 ± 1.8	45.5 ± 2.0	51.2 ± 2.9	45.3 ± 2.1	42.4 ± 2.8	46.8 ± 2.6	0.235	0.735	0.087
LDL	(mg 100 mL^−1^)	137.8 ± 6.0	143.6 ± 7.1	152.0 ± 6.1	124.8 ± 8.9 ^#^	144.5 ± 19.3	143.2 ± 6.5	0.524	0.093	0.247
Total cholesterol	(mg 100 mL^−1^)	203.4 ± 7.2	213.6 ± 7.0	217.8 ± 8.4	190.2 ± 10.4	223.6 ± 16.1	209.6 ± 7.6	0.178	0.015	0.810
Triglycerides	(mg 100 mL^−1^)	113.2 ± 12.1	156.9 ± 19.8	96.2 ± 11.6	128.9 ± 20.3	203.3 ± 44.5 *	138.4 ± 20.6	0.014	0.270	0.016
Homocysteine	(µmol L^−1^)	11.15 ± 1.22	8.92 ± 0.45	12.37 ± 2.46	10.04 ± 0.75	7.84 ± 0.70	9.37 ± 0.55	0.042	0.749	0.126
TNF	(pg mL^−1^)	8.88 ± 1.06	7.03 ± 0.29	8.67 ± 2.07	9.09 ± 0.67	7.44 ± 0.73	6.86 ± 0.27 **	0.111	0.863	0.572
cystine (plasma)	(µmol L^−1^)	50.6 ± 3.0	56.7 ± 2.3	41.0 ± 3.8	59.3 ± 3.0 ^##^	51.9 ± 4.6	58.6 ± 2.6	0.164	<0.001	0.207
cystine (PBMC)	(nmol mg^−1^)	n.d.	n.d.	n.d.	n.d.	n.d.	n.d.			
glutamate (plasma)	(µmol L^−1^)	51.7 ± 7.1	67.2 ± 5.1	29.0 ± 5.6	70.9 ± 9.4 ^##^	68.1 ± 13.4 **	66.8 ± 5.1	0.043	0.007	0.020
glutamate (PBMC)	(nmol mg^−1^)	150.0 ± 9.4	162.6 ± 10.6	153.3 ± 12.3	146.7 ± 14.8	135.3 ± 26.0	172.6 ± 10.5	0.791	0.188	0.081
glycine (plasma)	(µmol L^−1^)	173.0 ± 11.2	163.6 ± 7.5	160.7 ± 13.3	184.3 ± 17.8	169.4 ± 19.7	161.2 ± 7.2	0.564	0.508	0.297
glycine (PBMC)	(nmol mg^−1^)	22.8 ± 2.0	19.8 ± 1.1	25.4 ± 3.0	20.1 ± 2.4	16.1 ± 3.2	21.2 ± 0.9 ^#^	0.088	0.986	0.025
Total GSH	(nmol mg^−1^)	59.01	75.28	39.42	77.10	77.47	74.45			
Reduced GSH	(nmol mg^−1^)	48.87	67.43	34.20	64.42	69.04	66.81			
GSSG	(nmol mg^−1^)	9.09	7.86	5.22	12.67	8.43	7.64			
Red. GSH/GSSG	(ratio)	5.38	8.56	6.56	5.08	8.19	8.74			

Data represent mean ± SEM. A two-factorial ANOVA was applied to the total study population to detect a significant impact of OSA, obesity (OBS) or their interaction (see *p* values right side). Differences between groups or strata as detected by unpaired *t*-test (posthoc) are indicated as: * *p* < 0.05, ** *p* < 0.01, *** *p* < 0.001 OSA vs. control; ^#^ *p* < 0.05, ^##^ *p* < 0.01, ^###^ *p* < 0.001 obese vs. non-obese strata. Tu90% = time at O_2_-saturation < 90%; BP = brachial blood pressure, systolic or diastolic; IMT = intima-media-thickness, HOMA-IR = Homeostasis assessment model index of insulin resistance (see Methods), HDL/LDL = high-/low-density lipoprotein; n.d. = not detectable; GSH = glutathione, PBMC = peripheral blood mononuclear cells, GSSG = glutathione-disulfide (determined in pooled samples, data represent mean of 7 measurements).

**Table 2:** Cross-sectional comparison between a group with cumulative hypoxic stress (cHPX, time at O_2_-saturation < 90% > 15.2 min) and controls with stratification for non-obese and obese BMI: Anthropometry, polysomnography, vascular risk factors, cystine, as well as glutamate and glycine.

		Control	cHPX	Control		cHPX		*p* (ANOVA)		
		Total	Total	Non-Obese	Obese	Non-Obese	Obese	cHPX	OBS	Interact.
*n*		32	21	16	16	5	16	53	53	53
Age	(years)	45.9 ± 1.1	48.1 ± 1.6	45.1 ± 1.6	46.7 ± 1.5	54.0 ± 2.9 *	46.3 ± 1.7 ^#^	0.038	0.125	0.023
BMI	(kg m^−2^)	29.2 ± 0.8	32.7 ± 0.9 **	25.3 ± 0.6	33.1 ± 0.5 ^###^	27.0 ± 0.9	34.5 ± 0.7 ^###^	0.053	<0.001	0.842
AHI	(h^−1^)	11.8 ± 2.6	53.4 ± 5.4 ***	11.3 ± 3.1	12.3 ± 4.3	51.7 ± 9.6 ***	54.0 ± 6.6 ***	<0.001	0.795	0.915
Tu90%	(min)	2.4 ± 0.6	81.0 ± 16.7 ***	2.8 ± 1.1	1.9 ± 0.6	78.6 ± 23.3 *	81.8 ± 21.1 **	<0.001	0.941	0.896
Mean SaO_2_	(%)	94.4 ± 0.1	91.8 ± 0.4 ***	94.3 ± 0.2	94.5 ± 0.2	91.9 ± 0.5 ***	91.7 ± 0.5 ***	<0.001	0.976	0.686
Heart rate (at rest)	(min^−1^)	67.2 ± 1.8	71.1 ± 2.8	70.0 ± 2.8	64.3 ± 2.1	70.2 ± 7.7	71.4 ± 2.9 *	0.298	0.520	0.325
BP systolic	(mmHg)	138.6 ± 2.0	148.0 ± 3.0 *	132.9 ± 1.8	144.4 ± 3.1 ^##^	145.3 ± 7.7	148.8 ± 3.2	0.026	0.047	0.281
BP diastolic	(mmHg)	89.4 ± 1.4	95.1 ± 1.9 *	86.5 ± 1.6	92.3 ± 2.1 ^#^	92.2 ± 3.9	91.7 ± 0.5	0.067	0.059	0.690
carotid IMT	(mm)	0.62 ± 0.02	0.72 ± 0.03 **	0.60 ± 0.03	0.64 ± 0.02	0.79 ± 0.08 **	0.70 ± 0.03	0.001	0.539	0.107
HbA1c	(%)	5.50 ± 0.05	5.82 ± 0.10 **	5.54 ± 0.05	5.48 ± 0.08	5.72 ± 0.07	5.85 ± 0.13 *	0.015	0.770	0.363
Glucose (fasted)	(mg 100 mL^−1^)	92.1 ± 1.5	101.1 ± 2.2 ***	94.1 ± 2.5	90.2 ± 1.6	99.4 ± 3.7	101.6 ± 2.7 ***	0.005	0.773	0.289
Insulin (fasted)	(µU mL^−1^)	9.8 ± 2.1	14.6 ± 1.7	10.7 ± 3.9	8.9 ± 1.5	10.9 ± 2.9	15.8 ± 2.0 *	0.272	0.640	0.312
HOMA-IR	(---)	2.04 ± 0.48	3.67 ± 0.43 *	2.08 ± 0.93	2.00 ± 0.37	2.79 ± 0.81	3.94 ± 0.50 **	0.100	0.499	0.440
HDL	(mg 100 mL^−1^)	49.3 ± 1.7	42.8 ± 2.1 *	50.7 ± 2.2	47.8 ± 2.7	37.8 ± 2.6 **	44.5 ± 2.5	0.008	0.524	0.113
LDL	(mg 100 mL^−1^)	139.9 ± 4.9	142.3 ± 9.4	149.4 ± 5.5	130.5 ± 7.5	151.4 ± 30.7	139.2 ± 8.3	0.612	0.145	0.752
Total cholesterol	(mg 100 mL^−1^)	208.8 ± 6.2	208.9 ± 8.7	220.1 ± 8.3	197.5 ± 8.5	230.6 ± 21.9	202.1 ± 8.9	0.500	0.026	0.789
Triglycerides	(mg 100 mL^−1^)	130.0 ± 13.8	145.8 ± 22.8*	128.1 ± 22.0	131.9 ± 17.5	207.2 ± 59.4	126.6 ± 22.6	0.186	0.169	0.131
Homocysteine	(µmol L^−1^)	10.3 ± 1.0	9.3 ± 0.5	10.7 ± 1.9	9.9 ± 0.6	9.1 ± 0.8	9.3 ± 0.6	0.460	0.848	0.733
TNF	(pg mL^−1^)	8.18 ± 0.82	7.40 ± 0.41	8.16 ± 1.53	8.21 ± 0.58	8.20 ± 0.98	7.13 ± 0.43	0.661	0.665	0.637
cystine (plasma)	(µmol L^−1^)	50.1 ± 2.7	59.4 ± 2.0 **	40.9 ± 3.1	59.4 ± 3.1 ^###^	58.0 ± 4.4 *	59.8 ± 2.3	0.016	0.006	0.022
cystine (PBMC)	(nmol mg^−1^)	n.d.	n.d.	n.d.	n.d.	n.d.	n.d.			
glutamate (plasma)	(µmol L^−1^)	52.4 ± 6.1	71.3 ± 5.2 *	33.4 ± 6.3	70.2 ± 8.2 ^###^	80.3 ± 14.2 **	68.5 ± 5.3	0.011	0.148	0.006
glutamate (PBMC)	(nmol mg^−1^)	145.2 ± 8.1	173.5 ± 12.3	143.9 ± 10.4	146.8 ± 13.2	146.8 ± 46.3	180.2 ± 10.9	0.276	0.276	0.359
glycine (plasma)	(µmol L^−1^)	167.1 ± 9.8	169.4 ± 7.4	154.8 ± 11.2	179.3 ± 15.9	191.1 ± 23.1	162.6 ± 6.3	0.512	0.894	0.080
glycine (PBMC)	(nmol mg^−1^)	20.9 ± 1.6	21.8 ± 1.5	22.7 ± 2.6	18.8 ± 1.7	18.1 ± 5.6	22.7 ± 1.3	0.901	0.873	0.115

Data represent mean ± SEM. Note that because GSH and GSSG were determined in pooled samples according to OSA diagnosis 1, no data are presented for this explorative cHPX group assignment. A two-factorial ANOVA was applied to the total study population to detect a significant impact of cHPX, OBS, or their interaction (see *p* values right side). Differences between groups or strata as detected by unpaired *t*-test (posthoc) are indicated as: * *p* < 0.05, ** *p* < 0.01, *** *p* < 0.001 cHPX vs. control; ^#^ *p* < 0.05, ^##^ *p* < 0.01, ^###^ *p* < 0.001 obese vs. non-obese strata. Tu90% = time at O_2_-saturation < 90%; BP = brachial blood pressure, systolic or diastolic; IMT = intima-media-thickness, HOMA-IR = Homeostase assessment model index of insulin resistance (see Methods), HDL/LDL = high-/low-density lipoprotein; n.d. = not detectable.

**Table 3:** Pre- and post-CPAP values of BMI, sleep data, vascular risk factors, as well as plasma and PBMC amino acid and PBMC glutathione.

		OSA (AHI > 15 h^−1^)		cHPX (Tu90% > 15.2 min)	
		Pre-cPAP	Post-cPAP	Pre-cPAP	Post-cPAP
*n*		15	15	12	12
BMI	(kg m^−2^)	32.5 ± 1.3	32.7 ± 1.3	33.4 ± 1.4	33.7 ± 1.4
CPAP	(cm H_2_O)		7.80 ± 0.42		8.23 ± 0.41
AHI	(h^−1^)	47.9 ± 7.5	3.5 ± 1.4 §§§	52.1 ± 6.9	5.1 ± 1.6 §§§
Tu90%	(min)	73.0 ± 21.9	0.0 ± 0.0 §§	89.8 ± 25.1	0.0 ± 0.0 §§
Heart rate (at rest)	(min^−1^)	69.6 ± 2.5	65.4 ± 2.3	69.3 ± 3.0	63.8 ± 2.6
BP systolic	(mmHg)	148.0 ± 3.8	143.3 ± 2.6	150.8 ± 3.2	145.2 ± 3.0 §
BP diastolic	(mmHg)	93.8 ± 2.5	89.9 ± 2.0	94.9 ± 2.4	91.3 ± 2.4 §
carotid IMT	(mm)	0.68 ± 0.03	0.71 ± 0.04	0.69 ± 0.03	0.73 ± 0.04
HbA1c	(%)	5.78 ± 0.12	5.74 ± 0.12	5.79 ± 0.13	5.75 ± 0.12
Glucose (fasted)	(mg 100 mL^−1^)	102.3 ± 3.3	100.0 ± 3.3	103.4 ± 3.2	101.7 ± 4.0
Insulin (fasted)	(µU mL^−1^)	17.2 ± 3.2	13.1 ± 1.6	15.1 ± 2.3	13.0 ± 1.6
HOMA-IR	(---)	4.44 ± 0.93	3.21 ± 0.39	3.82 ± 0.54	3.22 ± 0.41
HDL	(mg 100 mL^−1^)	45.1 ± 2.6	47.4 ± 1.8	42.5 ± 2.4	45.2 ± 1.5
LDL	(mg 100 mL^−1^)	148.9 ± 12.2	148.6 ± 11.5	153.2 ± 14.7	151.3 ± 13.7
Total cholesterol	(mg 100 mL^−1^)	214.5 ± 11.5	216.5 ± 11.8	215.7 ± 13.9	215.0 ± 13.6
Triglycerides	(mg 100 mL^−1^)	148.0 ± 25.0	156.0 ± 27.2	143.1 ± 28.3	142.2 ± 23.8
Homocysteine	(µmol L^−1^)	8.50 ± 0.53	8.77 ± 0.45	8.65 ± 0.68	8.78 ± 0.52
TNF	(pg mL^−1^)	6.94 ± 0.35	7.12 ± 0.35	7.01 ± 0.44	7.24 ± 0.41
cystine (plasma)	(µmol L^−1^)	57.9 ± 3.0	49.8 ± 2.8	60.8 ± 2.7	48.1 ± 3.1 §§
cystine (PBMC)	(nmol mg^−1^)	n.d.	n.d.	n.d.	n.d.
glutamate (plasma)	(µmol L^−1^)	65.1 ± 5.1	50.1 ± 4.6 §	69.3 ± 5.8	49.3 ± 5.1 §§
glutamate (PBMC)	(nmol mg^−1^)	161.1 ± 13.6	144.7 ± 11.9	166.2 ± 16.3	137.9 ± 12.7
glycine (plasma)	(µmol L^−1^)	154.0 ± 9.1	134.1 ± 7.0 §	159.7 ± 5.9	138.8 ± 7.6 §
glycine (PBMC)	(nmol mg^−1^)	19.7 ± 1.6	17.2 ± 1.3	20.3 ± 2.0	16.5 ± 1.3
Total GSH	(nmol mg^−1^)	75.28	58.05		
Reduced GSH	(nmol mg^−1^)	67.43	51.81		
GSSG	(nmol mg^−1^)	7.86	6.25		
Red. GSH/GSSG	(ratio)	8.56	8.37		

Data represent mean ± SEM for a subgroup of OSA patients (AHI > 15 h^−1^) and the explorative cHPX subgroup (time at O_2_-saturation < 90% (Tu90%) > 15.2 min). Data on glutathione measurements were pooled for OSA patients. § *p* < 0.05, §§ *p* < 0.01, §§§ *p* < 0.001 by paired *t*-test comparing post- vs. pre-CPAP values.

## Data Availability

The underlying data set is available from the corresponding author upon reasonable request.
